# Decreased hippocampal neurogenesis and post-stroke depression

**DOI:** 10.3389/fpsyt.2026.1761408

**Published:** 2026-03-04

**Authors:** Xiangyue Tang, Lusen Ran, Wenfei Li

**Affiliations:** Department of Neurology, Shenzhen Nanshan People’s Hospital, Shenzhen, China

**Keywords:** Antidepressants, hippocampus, neurogenesis, neuroregulation, post-stroke depression, treatment

## Abstract

Post-stroke depression (PSD) is a common and serious complication following stroke, affecting approximately one-third of survivors and contributing to poor functional recovery. Its pathophysiology is multifactorial, with recent evidence highlighting decreased hippocampal neurogenesis as a key mechanistic contributor. This review aims to examine the specific role of hippocampal neurogenesis in PSD, focusing on (1) the evidence linking impaired neurogenesis to PSD onset (2), the underlying molecular and cellular mechanisms—including dysregulation of the hypothalamic–pituitary–adrenal(HPA) axis, neuroinflammation, altered neurotrophic signaling, and neurotransmitter disturbances—and (3) current and emerging therapeutic strategies that promote neurogenesis for PSD management. Pharmacological agents (e.g., antidepressants), neuroregulatory interventions, and lifestyle-based approaches show promise in restoring neurogenic activity and alleviating depressive symptoms. In conclusion, impaired hippocampal neurogenesis represents a central pathway in PSD pathogenesis, offering a valuable target for future research and therapeutic development. Further studies are needed to fully elucidate these mechanisms and translate neurogenesis-focused treatments into clinical practice.

## 1 Introduction

Stroke is a serious cerebrovascular condition that often leads to significant impairments in a patient’s physical, psychological, and social functioning. One of the common and serious sequelae following a stroke is post-stroke depression (PSD), which is characterized by a pervasive state of depression encompassing depressed mood, anhedonia, alterations in eating and sleeping patterns, fatigue, feelings of worthlessness, social withdrawal, and in severe cases, suicidal ideation (1, 2).

The clinical and epidemiological burden of PSD is substantial. Approximately one-third of stroke survivors may suffer from PSD in the first year post-stroke, with peak incidence often observed within the first three months (2–4). Between 25% and 50% of patients develop depression during the acute phase, of whom approximately 30% continue to suffer from it into the chronic phase (5). The incidence of PSD has been steadily increasing, with global prevalence estimates ranging from 27.5% to 62.5% (6). This condition is not merely a reactive psychological distress; it is independently associated with poorer functional recovery, increased cognitive impairment, higher mortality, and greater caregiver burden, thereby compounding the disability caused by the stroke itself (7).

The pathophysiology of PSD is considered multifactorial, arising from a complex interplay between ischemic neurobiological injury and psychosocial stressors. From a neurobiological perspective, research has increasingly focused on impaired adult hippocampal neurogenesis as a key etiological hypothesis.

In the adult mammalian brain, neurogenesis—the birth of new neurons—persists in two primary neurogenic niches: the subventricular zone (SVZ) of the lateral ventricles and the subgranular zone (SGZ) of the hippocampal dentate gyrus (DG). This review focuses on hippocampal neurogenesis, a multi-step process encompassing the proliferation of neural stem/progenitor cells (NSCs/NPCs), their differentiation into neuronal lineages, followed by maturation, migration, and functional integration into existing hippocampal circuits (8, 9). Within the SGZ, NSCs predominantly reside in a quiescent state. Once activated, these NSCs exit quiescence, re-enter the cell cycle, and initiate proliferation. The critical balance between the quiescent and active states is tightly governed by a complex interplay of intrinsic and extrinsic regulatory factors within the DG microenvironment. These factors include neurotrophic signals (e.g., BDNF), neurotransmitters (e.g., serotonin, glutamate), inflammatory cytokines, glucocorticoid levels, and experiential inputs such as environmental enrichment, learning, and stress (10).

Critically, adult hippocampal neurogenesis is now recognized as a fundamental component of neural plasticity that underpins specific forms of learning and memory, and, importantly, contributes to emotional regulation and stress resilience (11). A convergence of evidence from research has established that a decline in hippocampal neurogenesis is not merely a correlate but a contributing pathogenic mechanism in several neuropsychiatric and neurodegenerative disorders, most notably major depressive disorder (MDD), stroke, alzheimer disease and epilepsy (12–14). Preclinical studies demonstrate that suppressing neurogenesis can induce depressive-like behaviors, while enhancing it is a common, though not exclusive, mechanism of action for various antidepressant treatments and interventions (14).

The hippocampus is particularly vulnerable to a multitude of stressors relevant to the post-stroke state, including direct ischemic insult, secondary neuroinflammation, hyperactivity of the HPA axis, and psychosocial distress. Therefore, the significant reduction in hippocampal neurogenesis observed after stroke is posited to represent a critical convergence point where these diverse pathological pathways translate into the persistent depressive symptoms characteristic of PSD (12, 13).

This review will therefore explore the specific role of hippocampal neurogenesis in PSD by addressing the following key questions: (1) What is the evidence that decreased hippocampal neurogenesis contributes to the development of PSD? (2) What are the primary mechanisms through which a stroke and its sequelae lead to reduced hippocampal neurogenesis? (3) Based on this mechanistic understanding, what are the current and potential future treatment strategies for PSD that target neurogenic processes?

## 2 Decreased hippocampal neurogenesis induce PSD

### 2.1 Decreased hippocampal neurogenesis in stroke

A limited number of endogenous NSCs are enriched in the DG and SVZ. These NSCs possess the ability to self-renew and differentiate into neurons and various other cell types. The proliferation, differentiation, and survival of these NSCs are influenced by multiple factors within the DG microenvironment (15), including neurotransmitters, growth factors, cytokines, and hormones secreted by other cell types (16, 17). These processes are believed to play a crucial role in maintaining the structural and functional integrity of the hippocampus.

Ischemic injury enhances the proliferation of endogenous NSCs during the acute phase post-stroke (18, 19). However, this increase in NSCs does not appear to significantly impact the hippocampal formation (20–22). Several potential explanations may account for this phenomenon. Firstly, NSC proliferation is typically restricted to the acute and/or subacute phases of stroke. Kathner-Schaffert et al. demonstrated a significant decline in endogenous NSCs proliferation six months following a stroke (23). Generally, this proliferation begins approximately one week post-stroke, peaks around days 10 to 14, and subsequently returns to baseline levels within four to five weeks after onset (19, 24). Secondly, only a small fraction of endogenous NSCs are capable of surviving due to the inhospitable microenvironment in the post-stroke brain (19). Thirdly, the stroke event can lead to a shift in neurogenesis toward astrogenesis (25), which, in theory, reduces the pool of newly generated neurons. Furthermore, an increased number of morphologically altered aberrant neurons, characterized by aberrant basal dendritic trees or abnormal locations within the hilus, can be observed in the hippocampus following a stroke (19, 26). Consequently, while hippocampal neurogenesis is activated post-stroke, it may represent an abnormal process both functionally and structurally (27).

### 2.2 Decreased hippocampal neurogenesis and depression

Depression is a common mental health disorder characterized by complex and diverse pathological mechanisms. Numerous studies have demonstrated a significant reduction in neurogenesis within the hippocampus of patients with depression, which is closely associated with symptoms such as memory impairment and difficulties with concentration. Moreover, mouse models of major depressive disorder (MDD) show decreased proliferation and differentiation of neurons in the hippocampus (28–31).

The onset of depression is often influenced by a range of physiological and environmental factors, including chronic stress, inflammatory responses, and hormonal imbalances (32). Research has indicated that prolonged stress can lead to a decrease in neurotrophic factors in the hippocampus, such as brain-derived neurotrophic factor (BDNF). This reduction directly inhibits neurogenesis in the hippocampus, potentially exacerbating depressive symptoms (33). Furthermore, inflammatory responses are also believed to play a critical role in the pathophysiology of depression, with inflammatory markers identified as significant in the brains of individuals with depression, adversely affecting hippocampal structure and function, and further diminishing neurogenesis (34–36).

Interestingly, existing treatments for depression, such as antidepressant medications and psychotherapy, have been shown to promote neurogenesis in the hippocampus. For instance, selective serotonin reuptake inhibitors (SSRIs) enhance neurotransmitter transmission and facilitate neuronal growth by increasing the expression of BDNF. Consequently, these therapeutic approaches not only alleviate depressive symptoms but may also improve cognitive function in patients by enhancing neurogenesis within the hippocampus (8). These findings collectively suggest that decreased hippocampal neurogenesis may contribute to depressive symptoms.

### 2.3 Decreased hippocampal neurogenesis and PSD

Hippocampal neurogenesis plays a critical role in both the development of ischemic brain injury and depression. Indeed, accumulating literature has revealed the correlation between hippocampal neurogenesis and PSD (37, 38). Mariel Pietri et al. built a middle cerebral artery occlusion (MCAO) model of mice, which reliably replicates the core clinical features of stroke and associated PSD, including motor and cognitive deficits, neuronal loss, and impaired neurogenesis, alongside the observation of depressive-like behaviors ten weeks post-stroke (24).

It has been posited that PSD is a consequence of decreased hippocampal neurogenesis in stroke patients. However, due to the challenges in obtaining human hippocampal tissue samples, a reliable animal model is essential for further study. Moriyama et al. developed a PSD model by subjecting MCAO-treated mice to chronic mild stress (CMS), which resulted in reduced sucrose consumption (a marker of anhedonia) (39). Their findings indicate that CMS treatment impairs spontaneous neurogenesis in the DG approximately one month post-stroke, aggravates apoptotic injury, and disrupts circuit integration in mature neonatal cells, ultimately leading to the occurrence of PSD (40, 41). Additionally, Kim et al. found that MCAO mice subjected to CMS exhibited anxiety-like behavior, anhedonia, and despair-like symptoms (28). Zhang et al. established a PSD model using MCAO in conjunction with post-stroke isolated housing conditions, observing impairments in hippocampal neurogenesis at 3 days and memory function at 14 days post-stroke (42).

## 3 Mechanism of decreased hippocampal neurogenesis

### 3.1 Hyperactivity of hypothalamic-pituitary-adrenal axis

**Figure 1:**
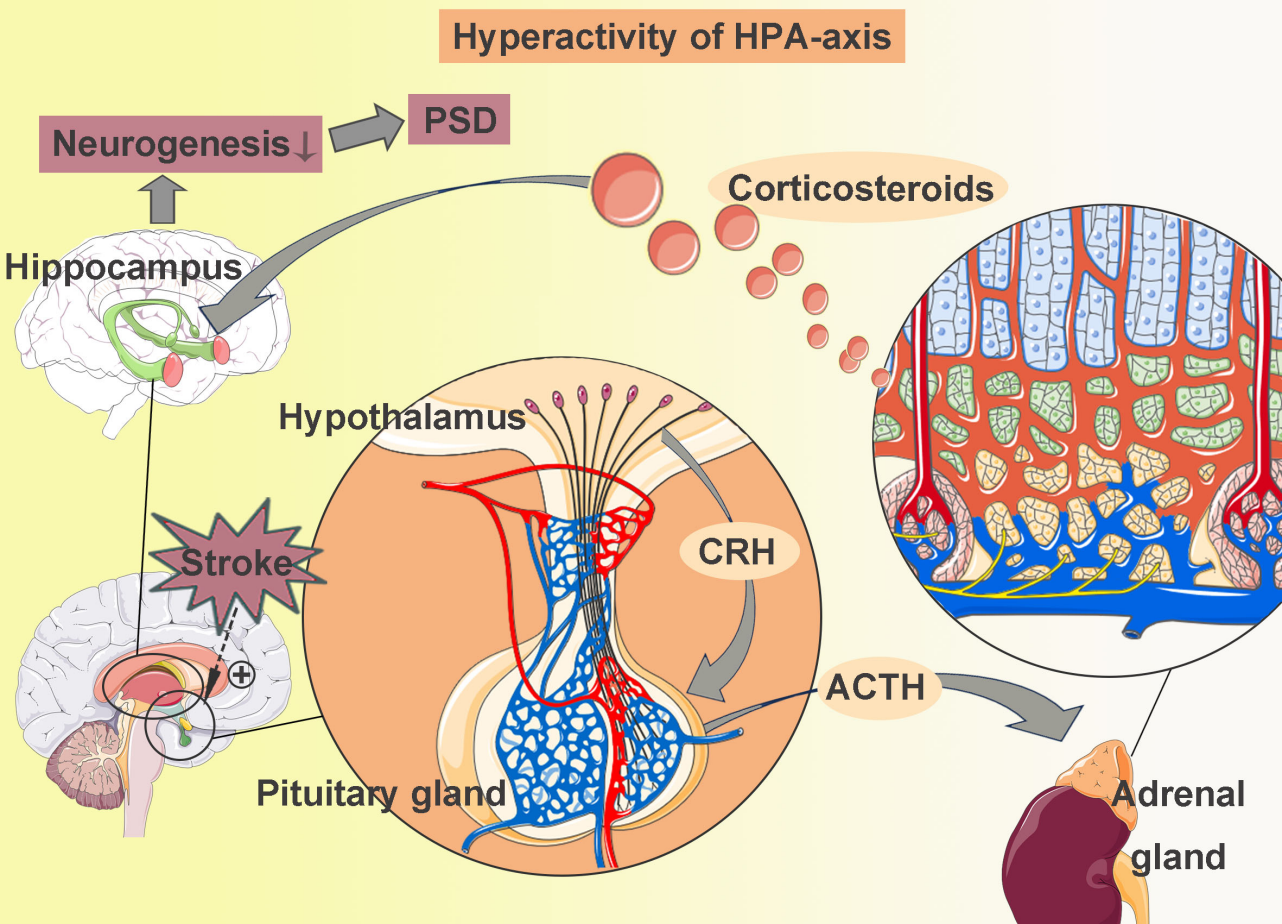
Hyperactivity of HPA-axis. When a stroke occurs, the hypothalamus receives signals of ischemic injury and sends them to the paraventricular nucleus. The paraventricular nucleus produces corticotropin-releasing hormone (CRH), which then stimulates the pituitary gland to release adrenocorticotropic hormone (ACTH). ACTH promotes the synthesis and release of corticosteroids. High corticosteroids levels could reduce hippocampal neurogenesis, thereby leading to post-stroke depression (PSD).

The hypothalamic-pituitary-adrenal (HPA) axis is a major neuroendocrine system responsible for the body’s response to stress. Hyperactivity of HPA axis is one of the most prominent biological findings in depression (43). Following a stroke, the hypothalamus detects ischemic injury signals and relays this information to the paraventricular nucleus, which subsequently produces corticotropin-releasing hormone (CRH). CRH stimulates the pituitary gland to release adrenocorticotropic hormone (ACTH), which promotes the synthesis and release of corticosteroids (44) (**Figure 1**). Elevated corticosteroid levels in the central nervous system (CNS) are significantly associated with PSD (45). Research has demonstrated that corticosteroids reduce neurogenesis and neuronal survival in the hippocampus (46).

The actions of corticosteroids are mediated by members of the nuclear receptor superfamily, specifically the glucocorticoid receptor (GR) and mineralocorticoid receptor (MR). These receptors bind to their ligands, functioning as transcription factors to directly regulate gene expression. They also produce rapid non-genomic effects on neuronal excitability and activation, thereby influencing cognitive processes (47, 48). In a study by Kim et al., a stress response was induced in mice using corticosterone, which resulted in reduced proliferation of hippocampal NSCs and NPCs, along with decreased expression of GR (49). Further confirmation of this association was supported through experiments involving GR siRNA knockdown and overexpression. Additionally, corticosteroids may act synergistically with neurotransmitters, neurotrophic factors, and other stress mediators, shaping both current and future responses to ischemic insults. Given its high density of GR and MR, the hippocampus is particularly sensitive to corticosteroid effects. Corticosteroids have been shown to decrease hippocampal neurogenesis and impair synaptic plasticity (46). Administration of the GR antagonist mifepristone can partially reverse PSD symptoms (50), further underscoring the importance of this pathway.

### 3.2 Neuroinflammation

Neuroinflammation occurs as a response to ischemic injury and involves a complex process characterized by the activation of inflammatory cells and the release of cytokines. Inflammatory mediators, such as tumor necrosis factor-alpha (TNF-α), interleukins (IL-1α, IL-1β, IL-6), and interferon (51), along with inflammatory cells like microglia and astrocytes, contribute to protecting the brain from further damage and promoting healing. However, excessive inflammation may lead to additional brain injury (52). The link between neuroinflammation and the onset of PSD has been well documented (53, 54).

**Figure 2:**
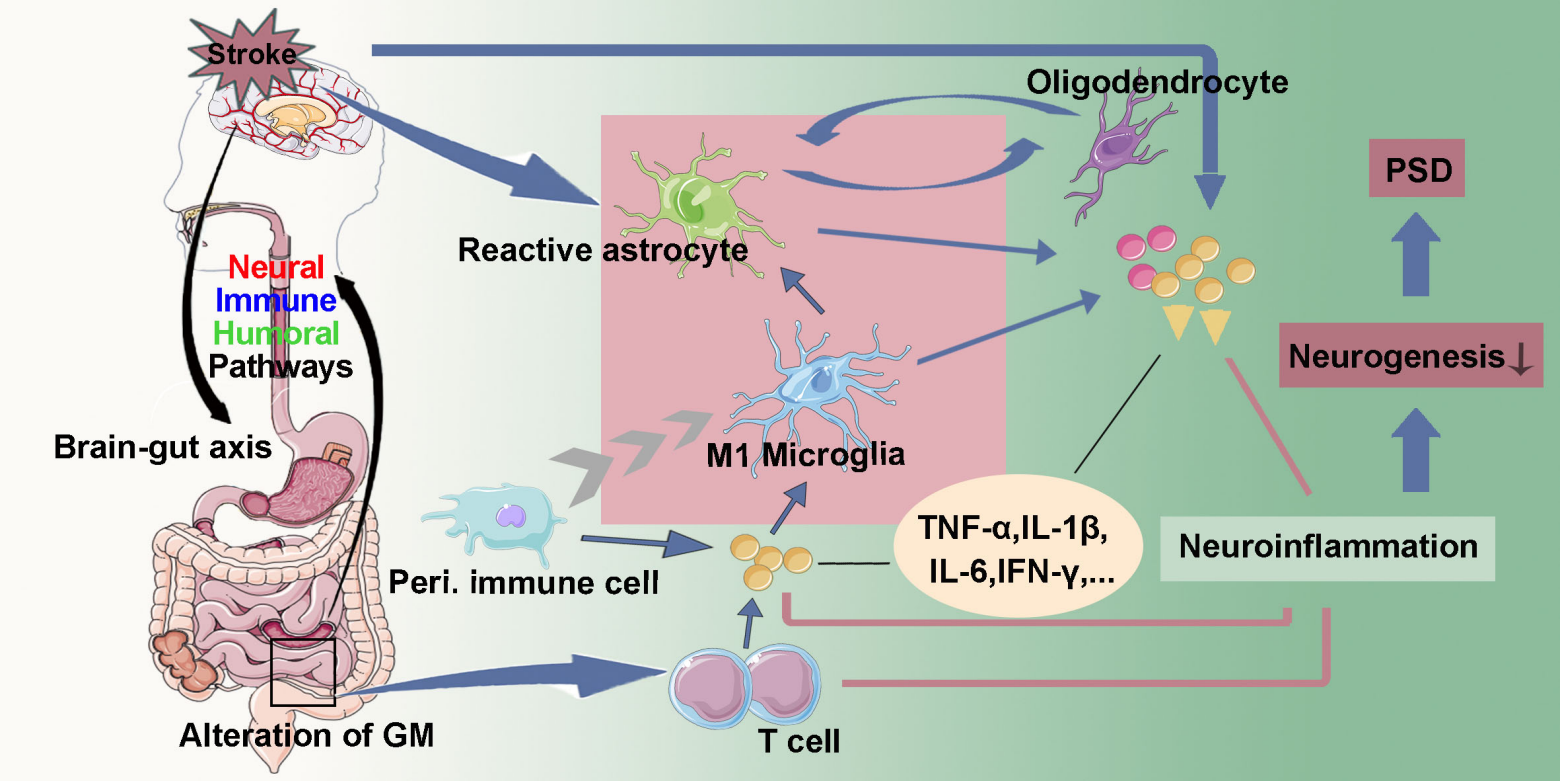
Neuroinflammation. After a stroke, activated microglia and astrocytes release inflammatory cytokines (TNF-α, IL-1β, IL-6, IFN-γ…). Pro-inflammatory (M1) microglia can exacerbate the neuroinflammatory response by recruiting peripheral immune cells and activating astrocytes. Oligodendrocytes may also play a role in neuroinflammation through their interactions with reactive astrocytes. Furthermore, ischemic injury signals may disrupt the gut microbiota (GM) via the gut-brain axis, resulting in increased intestinal permeability and triggering pro-inflammatory responses by altering T cell homeostasis. Neuroinflammation could reduce hippocampal neurogenesis, thereby leading to post-stroke depression (PSD).

After a stroke, microglia and astrocytes quickly recognize ischemic stimuli and neuronal damage, triggering an inflammatory response. Activated microglia and astrocytes release pro-inflammatory or anti-inflammatory factors, thus modulating neuroinflammation (52). Pro-inflammatory (M1) microglia can exacerbating neuroinflammation by recruiting peripheral monocytes and activating astrocytes (55). Inhibiting neuroinflammation has been shown to improve neurogenesis in animal models (56, 57). Additionally, CMS can promote pro-inflammatory differentiation of microglia and increase astrocyte activation in the DG while simultaneously inhibiting neurogenesis (58). Reactive astrocytes not only participate in neuroinflammation but also play a significant role in regulating neurotrophic factors expression and neuronal transmission (52, 59). Interestingly, oligodendrocytes may also be involved in neuroinflammation through communication with reactive astrocytes (60). Furthermore, neuroinflammation induced by abnormal peripheral immune cell activation can impair neurogenesis (35) (**Figure 2**).

Dysbiosis of intestinal microbiota has emerged as a recent area of interest concerning the mechanisms underlying PSD. Some researchers propose that stroke alters gut microbiota (GM), leading to increased gut permeability and the subsequent release of systemic inflammatory factors. This process may exacerbate the severity of stroke-related damage and raise the likelihood of post-stroke complications by disrupting T cell homeostasis, inducing pro-inflammatory responses, and promoting oxidative stress (61–63) (**Figure 2**). However, whether GM dysbiosis is a direct causal driver of impaired hippocampal neurogenesis and depressive symptoms—or rather a secondary consequence of stroke-related physiological stress—has yet to be definitively established. Although preclinical interventions such as probiotic supplementation and fecal microbiota transplantation have shown promising antidepressant effects, their clinical applicability, mechanistic specificity, and long-term safety remain unresolved.

Pro-inflammatory cytokines play a crucial role in the regulation of cell apoptosis and necrosis, particularly in the vulnerable hippocampus (40, 64, 65). Accumulating evidence suggests that inflammation promotes the development of PSD (52, 66–68). Various cytokines have been associated with reduced hippocampal neurogenesis (69, 70). Ischemic insults increase TNF-α levels, which adversely affect hippocampal neurogenesis, while exogenous TNF-α administration exacerbates cell proliferation and survival within the hippocampus (71). Moreover, IL-1β can have both positive and negative effects on hippocampal neurogenesis, depending on concentration and context. Low to moderate levels of IL-1β enhance NSC proliferation and survival, whereas excessive IL-1β exerts detrimental effects. Clinical research indicates that PSD patients present with elevated serum IL-1β levels compared to non-PSD patients (72). Furthermore, increased serum IL-6 levels in the acute phase of ischemic stroke have been independently associated with the onset of depression two weeks and one year post-stroke (66). Currently, it is known that IL-6 and IFN-γ have regulatory effects on the tryptophan-serotonin pathway (73).

### 3.3 Reduction of neurotrophic factors

Neurotrophic factors play an indispensable role in the proliferation, survival, development, function, and plasticity of neurons. Key neurotrophic factors include nerve growth factor (NGF), BDNF, neurotrophin-3/4, neurotrophin-5, and neurotrophin-6 (74). Additionally, several cytokines, such as leptin, insulin-like growth factor-1 (IGF-1), transforming growth factor, fibroblast growth factor, and platelet-derived growth factor (75), are present in the central nervous system and can modulate neurogenesis. Among these, BDNF is considered to have the most significant and widespread neurotrophic effect, particularly in promoting and sustaining neurogenesis in the hippocampus. BDNF not only supports the survival and differentiation of newly generated neurons but also facilitates their integration into existing neural circuits. Ischemic injury typically increases BDNF expression (76, 77). However, studies have reported that plasma levels of BDNF in patients with acute stroke are 2.5 times lower than those in healthy controls. Furthermore, the level of BDNF decreased in the chronic stage (78). Research by Mayuri N. Tuwar et al. indicated that patients with acute ischemic stroke had low BDNF levels upon admission (average 2.94 ng/mL) within the first 24 hours. These levels significantly increased to an average of 6.79 ng/mL between 25 and 48 hours but subsequently declined to 2.77 ng/mL after 96 hours (79).

**Figure 3:**
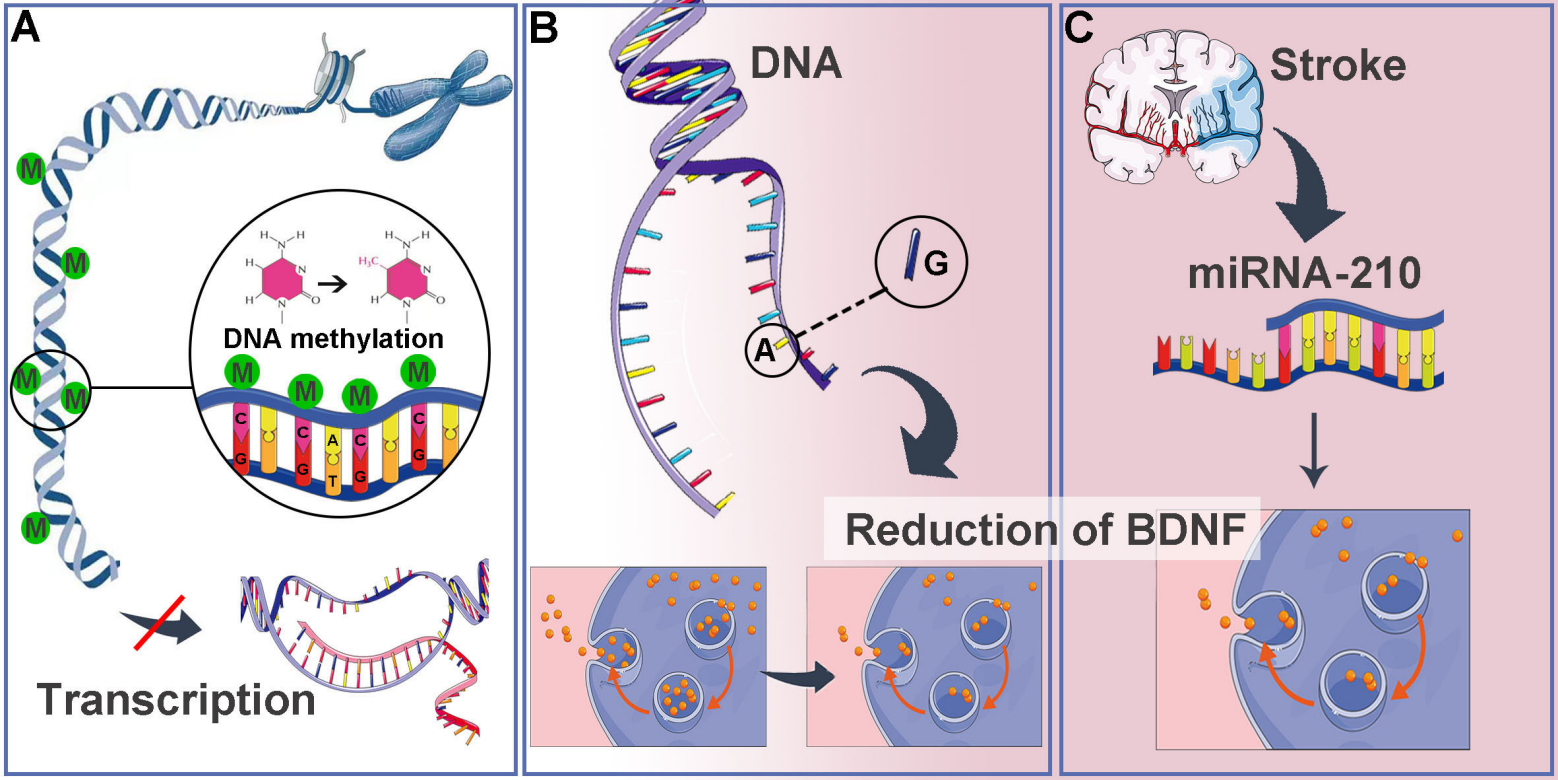
Reduction of BDNF. **(A)** Hypoxia induced hypermethylation of the BDNF gene contributes to the epigenetic silencing of CpG islands, resulting in decreased BDNF expression. **(B)** The BDNF SNP rs6265 replaces the G allele at position 196 of the BDNF gene with an A allele, leading to the conversion of the amino acid valine at codon 66 to methionine, which in turn reduces BDNF secretion. **(C)** Following a stroke, the expression of miRNA-210 is elevated, leading to gene silencing that affects BDNF expression.

DNA methylation, a process known to regulate gene expression at the transcriptional level, may contribute to the reduction of BDNF following a stroke (80, 81) (**Figure 3A**). Hypoxia-induced hypermethylation of the BDNF gene can lead to the epigenetic silencing of CpG islands, which may correlate with the prevalence and persistence of PSD (82). The application of 5-Aza-2′-deoxycytidine (a DNA methyltransferase inhibitor) after stroke has shown to increase BDNF levels by modulating DNA methylation (83). Additionally, single nucleotide polymorphisms (SNPs), such as the rs6265 variant, alter the BDNF gene from a G allele to an A allele at position 196, resulting in the amino acid substitution of valine at codon 66 with methionine (84, 85). This SNP is associated with a reduction in BDNF secretion without affecting overall gene expression (86) (**Figure 3B**). MiRNA-210 has been identified in cellular experiments as a regulator of BDNF transcription and is observed at elevated levels in stroke patients, negatively correlating with BDNF concentrations (**Figure 3C**).

Yang et al. demonstrated that serum BDNF concentrations below 5.86 ng/mL on day 1 post-stroke were independently correlated with PSD, with an 11.5-fold increase in PSD risk at three months when BDNF levels fell below 10.2 ng/mL (87). A multicenter prospective study conducted in 2024 revealed that elevated serum BDNF levels in ischemic stroke patients were independently associated with a reduced risk of developing PSD at three months (88). Chen et al. confirmed that low BDNF concentrations could lead to diminished neurogenesis rates in the hippocampus (89). Results from the study by Sheng Huang et al. indicate that the intravenous injection of hippocampal exosomes derived from stroke mice exacerbates depression-like behaviors in recipient mice. This effect is potentially mediated through the modulation of neurogenesis by depression-related proteins, specifically proBDNF and its receptor p75NTR (90). Maintaining low BDNF levels post-stroke may compromise the survival, differentiation, and integration of new neurons derived from NSCs in the hippocampus.

### 3.4 Disorder of neurotransmitter

**Figure 4:**
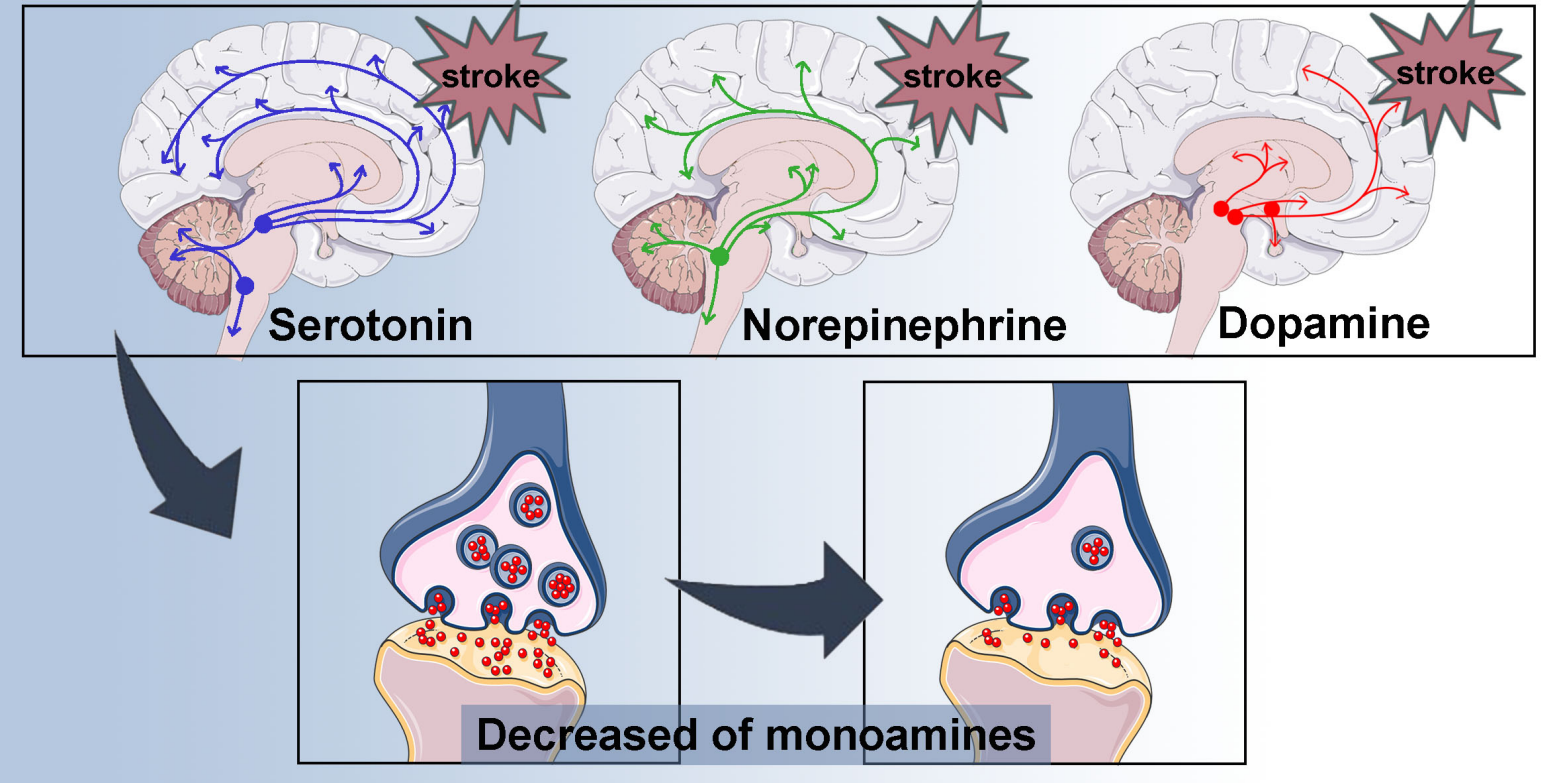
Decreased of monoamines. Cerebral ischemic lesions interrupt the projections ascending from midbrain and brainstem, passing through thalamus and basal ganglion, and reaching the frontal cortex, resulting in reduced synthesis of monoamines.

Neurotransmitters, chemical substances released from presynaptic neurons, bind to receptors on postsynaptic neurons to modulate various neural functions. Key neurotransmitters involved in the development of PSD include glutamate, dopamine, 5-HT, and norepinephrine. Among these, the monoamines-dopamine, 5-HT, and norepinephrine are found to be reduced in patients experiencing PSD (91). The amine hypothesis, which supplements the lesion location hypothesis, suggests that cerebral lesions disrupt projections ascending from the midbrain and brainstem through the thalamus and basal ganglia, ultimately reaching the frontal cortex, thereby reducing monoamine synthesis (**Figure 4**). Additionally, the disorder of 5-HT may be linked to DNA methylation of the serotonin transporter gene SLC6A4, which plays a critical role in 5-HT recycling (92). Highly methylated SLC6A4 promoters have been associated with depression following a stroke, from two weeks to more than one year (92).

5-HT functions as a growth-promoting factor in the central nervous system during development and is essential for neuronal and synaptic plasticity in the adult brain (93). Furthermore, proliferation of NPCs and survival of adult neurons are positively correlated with 5-HT levels (11). As emotional behaviors are regulated by several neurotransmitters, particularly monoamines, the symptoms of depression may result from impaired monoamine function (94). The concentration of monoamines is significantly lower in patients with depression (95), a similar trend observed in those with PSD (91). Of note, Wang et al. found a significant decrease in 5-HT1A receptors and mRNA levels in the hippocampus of animals exhibiting PSD (96). Hence, we speculate that the reductions in monoamines and/or their receptors in the hippocampus may lead to a reduction in neurogenesis, which and then triggers the PSD. Research by Villa et al. indicates a significant reduction in dopamine levels in rats exhibiting depression-like states, with this change closely associated with decreased functionality of D2 receptors (97). Dopamine functions as a neurotransmitter in the hippocampus, paralleling the role of the 5-HT system in regulating hippocampal neurogenesis, which contributes to depressive-like behaviors (12, 98). The noradrenergic system is believed to have a direct impact on hippocampal neurogenesis. Notably, the multi-step process of hippocampal neurogenesis, which includes proliferation, maturation, and survival, is positively regulated by the serotonergic system, while only the early stages of adult neurogenesis are influenced by the noradrenergic system (11).

Glutamate-mediated excitotoxicity may present another factor contributing to reduced hippocampal neurogenesis. Following an acute stroke, glutamate levels in both the brain and plasma increase (99). In patients with depression, cerebrospinal fluid concentrations of glutamate are significantly higher compared to healthy controls (100). A magnetic resonance spectroscopy (MRS) study (101) has yielded similar findings in patients with PSD. As a primary neurotransmitter in the brain, glutamate plays a crucial role in various brain functions, including neurogenesis and mood regulation. Evidence suggests that glutamate and the activation of its receptors significantly contribute to neurogenesis (102). Conversely, abnormally high levels of glutamate, resulting from increased release and/or decreased clearance, can cause neuronal atrophy, inhibit neurogenesis, and lead to depression (103). These opposing effects on neurogenesis and neuronal survival depend on glutamate concentration. An abnormal increase in glutamate following a stroke may inhibit neurogenesis in the hippocampus and remodel dendrites and cytoarchitecture (104, 105), consequently contributing to the development of PSD.

### 3.5 Crosstalk of cytokines, glucocorticoids, neurotransmitters and neuronutrients

The post-stroke neurobiological alterations do not occur in isolation. Complex “crosstalk” exists among the HPA axis, neuroinflammation, neurotransmitter systems and neuronutrients, collectively contributing to hippocampal neurogenesis impairment.

#### 3.5.1 The vicious cycle between inflammation and the HPA axis

Pro-inflammatory cytokines (e.g., IL-1β, IL-6, TNF-α) released after stroke are potent stimuli for HPA axis activation, leading to increased secretion of CRH and ACTH. This disrupts the normal negative feedback loop, resulting in sustained HPA axis dysfunction and elevated glucocorticoid levels (54, 106). While glucocorticoids are generally anti-inflammatory, their chronic excess in the post-stroke context can paradoxically induce pro-inflammatory gene expression in brain regions like the hippocampus and further modulate cytokine release from macrophages (107, 108). Simultaneously, microglia express corticosteroid receptors, making their function subject to this hormonal regulation (109). Consequently, inflammation and HPA axis hyperactivity mutually exacerbate each other, forming a vicious cycle that is difficult to break. Additionally, the stroke event itself acts as a potent stressor that may directly activate the HPA axis, although the primary initiating role of cytokines versus glucocorticoids in PSD pathogenesis remains a topic of ongoing investigation (110).

#### 3.5.2 A dual assault on neurotransmitter systems

The dysregulated inflammation-HPA axis nexus jointly impairs key neurotransmitter systems, particularly the serotonergic system. Neurotransmitter receptors may mediate pro-inflammatory signaling pathways, and CMS can exacerbate the pro-inflammatory state in the brain by disrupting the balance of these pathways involved in neuronal homeostasis (111).

- Direct Interference with Synthesis: Pro-inflammatory cytokines can potentially interfere with the synthesis and secretion of monoamine neurotransmitters (111–114). For instance, cytokines, especially IL-6, can up-regulate the expression of the enzyme indoleamine 2, 3-dioxygenase (IDO). IDO shunts tryptophan—the essential precursor for serotonin (5-HT)—away from the 5-HT synthesis pathway toward alternative metabolites. This directly leads to reduced central 5-HT availability and the production of potentially neurotoxic catabolites (115–117).
- Impact on Precursors and Transport: Hypercortisolism may upregulate tryptophan decarboxylase, depleting precursors for both 5-HT and norepinephrine (118), and has been linked to reduced thalamic 5-HT transporter levels. Genetic factors (e.g., the 5-HTTLPR polymorphism) may also mediate this process by influencing individual HPA axis reactivity to stress (119, 120).

#### 3.5.3 Convergence on neurotrophic factors

- The interaction between neuroinflammation and neurotrophic factors establishes a critical self-reinforcing cycle. Neuroinflammation can suppress the expression of BDNF by enhancing the methylation of the BDNF gene (121), a downregulation evidenced in animal models by LPS administration (122), and clinically correlated with PSD (82). Conversely, under PSD conditions, increased binding of the BDNF precursor (proBDNF) to its p75NTR receptor activates the RhoA/JNK signaling pathway (123). This promotes NF-κB translocation and pro-inflammatory gene transcription, leading to the release of additional inflammatory factors that further exacerbate neuroinflammation (124).
- Excess glucocorticoids can also suppress hippocampal BDNF expression (125). The reduction in BDNF weakens its crucial support for neuronal survival, synaptic plasticity, and neurogenesis.
- Serotonergic neurons themselves express BDNF, and serotonin transmission modulates the synthesis and release of BDNF, indicating a positive feedback relationship (126, 127). Therefore, decreased serotonin further exacerbates BDNF deficiency.

#### 3.5.4 Additional interactive factors

A meta-analysis indicates that patients with PSD exhibit significantly higher levels of serum leptin during the acute, subacute, and chronic phases compared to non-PSD patients (128). Its pathogenesis may be closely linked to the HPA axis, where axis overactivity promotes the release of adiponectin from adipose tissue, which in turn further enhances basal cortisol secretion, creating another reinforcing loop (129).

In conclusion, the interplay among monoamines, neuroinflammation, the HPA axis, and neuronutrients following a stroke culminates in disturbances in hippocampal neurogenesis. Understanding this interactive network is crucial for developing multi-target therapeutic strategies.

### 3.6 Chronic mild stress

**Figure 5:**
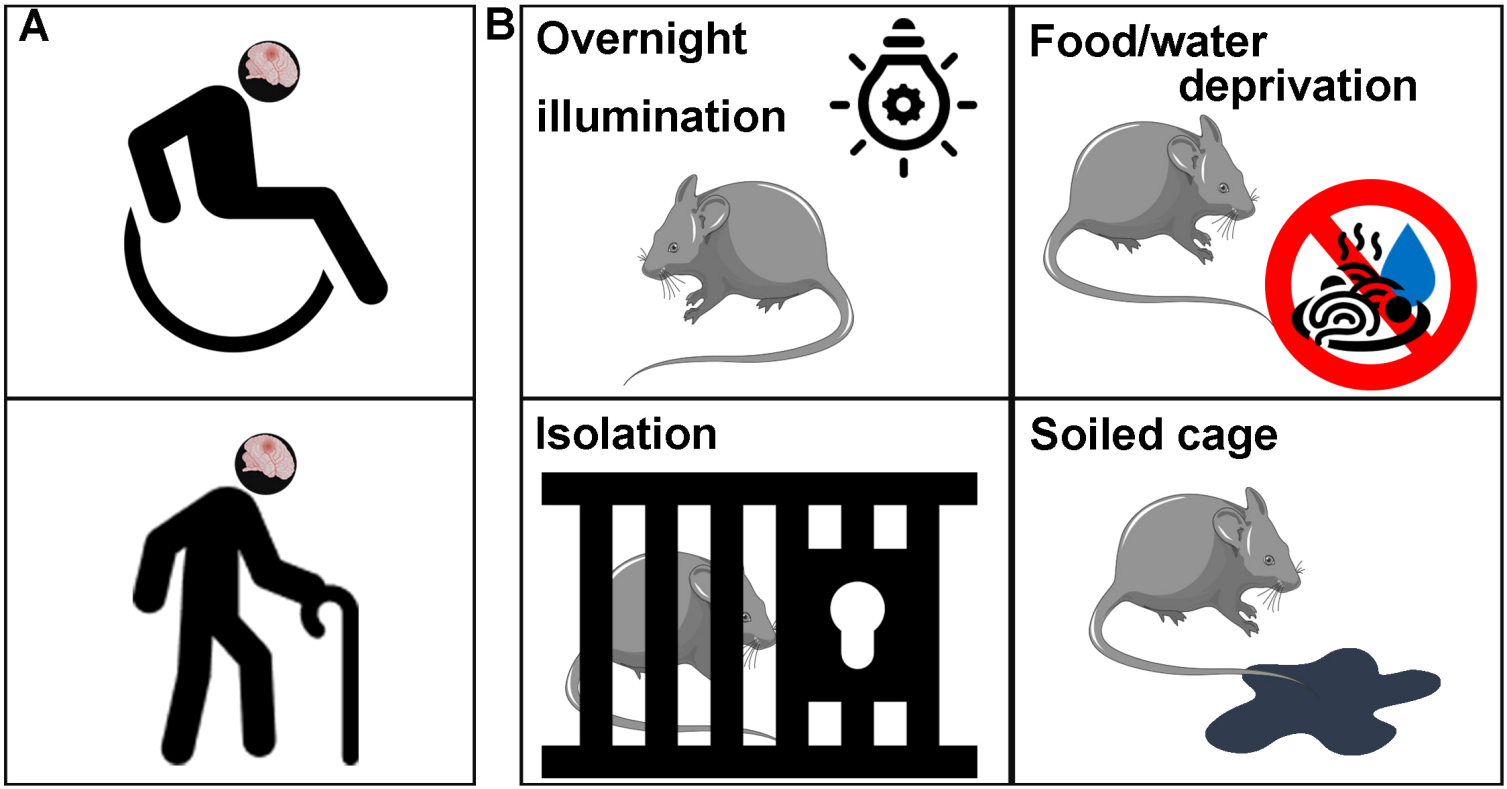
Chronic mild stress (CMS). **(A)** The social and psychological reactions caused by post-stroke functional impairments in patients. **(B)** The modified CMS programs in animal models include overnight illumination, food/water deprivation, social isolation and soiled cage, among others.

Aside from neurobiological factors, the social and psychological responses to post-stroke functional impairments are considered major contributors to PSD. Clinical researches have shown a higher risk of PSD with post-stroke functional impairments and lack of social support, both perceived and objective (130, 131) (**Figure 5A**). Wang et al. investigated predictors of PSD, revealing that neurological function had the most significant overall effect on PSD, with social support acting as the most substantial direct factor, while family function had the largest indirect effect (132). A cross-sectional study indicated a negative correlation between self-efficacy in participation and depression in stroke survivors (133). Increasing social support interventions would prevent or alleviate depressive symptoms (134).

Animal models of PSD are generally constructed by stroke model combined with CMS (28). The design of this combination includes mild or low-grade stressors, such as overnight illumination, food/water deprivation, social isolation and soiled cage (**Figure 5B**). These stressors aim to simulate the daily problems and difficulties faced by stroke patients. The modified CMS program has been widely recognized as an effective method to induce depression. The evaluation of depression and despair behaviors in these animals is conducted through sucrose consumption tests, forced swimming tests, and field trials (40, 94). CMS can exacerbate tissue damage following a stroke, leading to severe clinical symptoms of depression through mechanisms like neuronal loss, brain degeneration, and impaired neurogenesis (135, 136). In preclinical experiments, Zhang et al. found that chronic stress results in the depletion of the aNSC pool and impairs the development of new neurons in the DG (30). Sylvia F Fawzi et al. observed that norepinephrine and 5-HT levels were reduced in CMS rats, accompanied by a significant increase in corticosterone, leading to increased production of pro-inflammatory cytokines (IL-6 and TNF-α) (137). Additionally, CMS can also alter ischemia-induced neurogenic fate by promoting the differentiation of NPCs into glial lineage cells (41) with the Notch signaling pathway, which is especially expressed in sites of neurogenesis, including SGZ (138). This pathway affects the fate of NSCs, promoting differentiation into astrocytes while inhibiting differentiation into neurons and oligodendrocytes (139). Moreover, it affects the growth and extension of axons (140) and disrupts synaptic formation between mature and newborn neurons (141–143). Zhang et al. found that post-stroke social isolation-mediated PSD may reduce hippocampal neurogenesis and impair memory function, involving regulatory expression of transforming growth factor-β (42). Intranasal delivery of hyperforin alleviates the PSD by increasing hippocampal neurogenesis (42).

**Figure 6:**
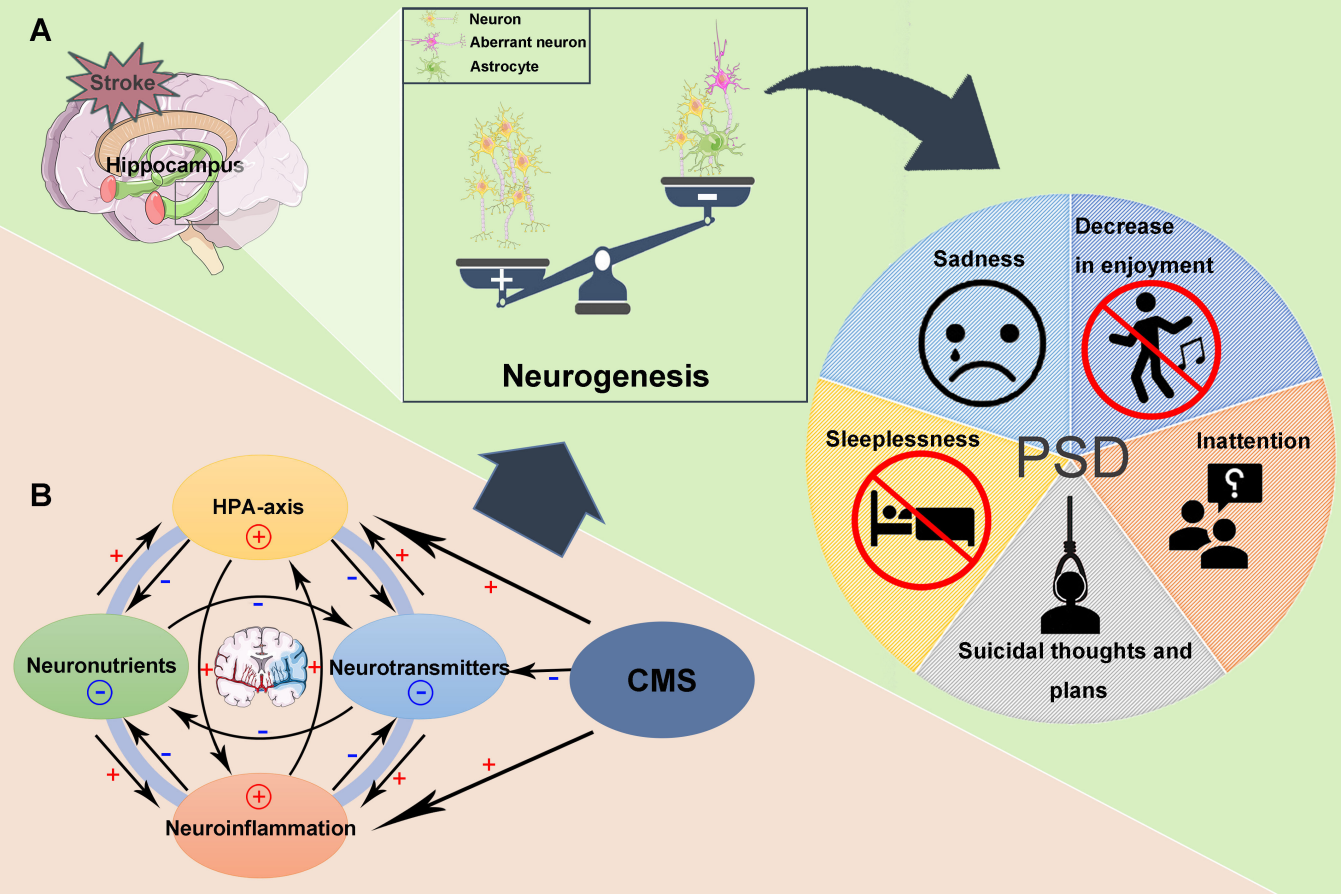
Decreased Hippocampal Neurogenesis and Post-Stroke Depression (PSD). **(A)** Following a stroke, reduced hippocampal neurogenesis can contribute to the onset of PSD. **(B)** After a stroke, hyperactivity of the hypothalamic-pituitary-adrenal (HPA) axis, reductions in monoamines, decreased levels of neurotrophic factors, neuroinflammation, and their interactions all contribute to diminished hippocampal neurogenesis. Chronic mild stress (CMS) further exacerbates this process by modulating the HPA-axis, promoting neuroinflammatory responses, and reducing intersynaptic monoaminergic neurotransmitter levels, thereby facilitating disease progression.

Studies have provided support for the close relationship between hippocampal neurogenesis and depression in adult mammals (32, 33). A decrease in proliferating and differentiated neurons has been observed in the hippocampus of depressed mice, a finding that is also noted in PSD models (28, 41). However, the specific mechanisms governing this process are not yet fully understood. It is currently widely believed that this relationship involves an interplay of neurobiological, behavioral, and social factors, including interactions among glucocorticoids, cytokines, neuronutrients, and neurotransmitters, alongside CMS-induced stress, leading to reduced hippocampal neurogenesis (144) (**Figures 6A, B**). These findings highlight the causal relationship between ischemia-induced neurogenesis and depression-like behavior, shedding light on the etiology of PSD and offering potential targets for treatment.

## 4 Treatments of PSD based on hippocampal neurogenesis

The neurogenesis hypothesis underscores the critical role of hippocampal neurogenesis in emotional regulation and the mechanism of antidepressant therapies. This section reviews and critically evaluates current and emerging interventions for PSD that are linked, either directly or indirectly, to the modulation of hippocampal neurogenesis, organizing them into pharmacological, non-pharmacological, and experimental/future directions.

### 4.1 Pharmacological interventions

Pharmacotherapy remains the cornerstone of PSD management. Several drug classes have demonstrated efficacy, with their mechanisms increasingly linked to promoting neurogenesis.

#### 4.1.1 Antidepressants

Antidepressants have been reported to improve motor, cognitive, and functional outcomes in individuals. The major antidepressants approved by the Food and Drug Administration (FDA) for the treatment of PSD include tricyclic antidepressants, SSRIs and serotonin-norepinephrine reuptake inhibitors (SNRIs) (145). These medications have been demonstrated to stimulate neurogenesis in the DG of adult rodents and non-human primates (11). Other drugs that have been shown to work are norepinephrine and dopamine reuptake inhibitors (NDRI), noradrenergic and specific serotonergic antidepressants (NaSSAs) and monoamine oxidase inhibitors (MAOI) (146). Among them, SSRIs are considered the most effective antidepressants (147), selectively inhibiting the reuptake of 5-HT by presynaptic membranes (148). Growing evidence indicates that the pharmacological activity of SSRIs is related to neuroplasticity, including promoting neurogenesis and synaptic transmission, partially mediated by activating neurotrophic factor signaling pathways, reducing oxidative stress and neuroinflammation (149–153). Additionally, antidepressant-induced neurogenesis appears to upregulate the expression of several key genes related to the vascular endothelial growth factor (VEGF), fibroblast growth factor (FGF), insulin-like growth factor (IGF), and other associated factors (11). Common SSRIs include fluoxetine, paroxetine, fluvoxamine, sertraline, citalopram and escitalopram. A meta-analysis of ten randomized controlled trials involving a total of 5370 patients demonstrated that early SSRIs therapies were effective to prevent PSD. Aside from increasing the risk of seizures and nausea, SSRIs are relatively safe (154). Another meta-analysis revealed that participants receiving SSRIs treatment had a lower risk of developing PSD, especially after one year (155). Escitalopram has been shown to regulate anti-inflammatory mediators, facilitate the regeneration of hippocampal neurons, and modulate the activity of the HPA axis, all of which are linked to improved treatment outcomes for depression (153, 156). Sertraline support neuronal survival and function by significantly improving BDNF expression (3). Fluoxetine exerts its pharmacological effects through activating multiple mechanisms, such as inducing microglial apoptosis (157), regulating the inflammatory processes (36, 148), enhancing neuroplasticity. Its induction of synaptic remodeling in hippocampal CA1 region promotes the release of BDNF and improve the efficiency of retrograde BDNF transport from CA3 to DG, thus triggering synaptic plasticity and activating cell survival mechanisms (158). A study using fluoxetine to treat cerebral ischemia in rats after NSCs transplantation showed that fluoxetine increased 5-HT levels and promoted neuronal differentiation (159). SSRIs can also stimulate neuronal generation and the secretion of growth factors, thus improving the prognosis of stroke (160, 161).

Due to the higher number of side effects associated with SSRIs, they are gradually being replaced by drugs with fewer adverse reactions and better tolerability, such as NaSSAs. The representative drug of NaSSAs is mirtazapine, which increases the release of 5-HT by directly inhibiting the α2 receptors at the terminals of 5-HT neurons, and further enhances the release of 5-HT by stimulating the α2 receptors on the cytoplasm of 5-HT neurons through increasing norepinephrine content. A network meta-analysis incorporating 51 randomized controlled trials evaluated the efficacy ranking of 9 antidepressants (sertraline, escitalopram, venlafaxine, paroxetine, duloxetine, amitriptyline, desipramine, trazodone, and mirtazapine) for the treatment of PSD. Results indicated that escitalopram ranked highest, while amitriptyline was the least helpful. At 4 weeks, sertraline ranked higher than placebo and the other eight antidepressants. At 8 weeks, mirtazapine ranked higher than sertraline and escitalopram. At endpoint, mirtazapine was associated with the highest remission rate, followed by venlafaxine and escitalopram (162). It is worth noting that the latest systematic review and meta-analysis of a randomized controlled trial indicate that the melatonin receptor agonist agomelatine can synergistically regulate the melanocortin pathway and the 5-HT pathway, stabilizing neurotransmitter networks. Its therapeutic effect on PSD may be comparable to SSRIs/SNRI and may potentially improve stroke outcomes with better safety (163). Selegiline, a monoamine oxidase inhibitor, enhances dopaminergic neurotransmission in the hippocampus and reduces damage associated with long-term potentiation, thereby improving depressive behaviors (37). Moreover, sertraline has been found to attenuate the degeneration of DA neurons and reduce depressive-like behaviors in rodents subjected to cerebral artery occlusion and reperfusion (164).

#### 4.1.2 Adjunctive and neuroprotective agents

Beyond classical antidepressants, a growing body of evidence suggests that adjunctive agents targeting specific neuropathological mechanisms in PSD possess neuroprotective and pro-neurogenic potential, offering complementary or alternative therapeutic strategies.

- Anti-inflammatory agents: A meta-analysis concluded that anti-cytokine therapy can improve depressive symptoms by promoting neurogenesis (165). Anti-inflammatory drugs increase the concentration of monoaminergic neurotransmitters in the synaptic cleft between neurons in the brain for a short period of time. In stroke patients, the use of acetylsalicylic acid, non-steroidal anti-inflammatory drugs, or statins reduced the risk of early-onset depression (166). Additionally, minocycline, a tetracycline antibiotic, has shown efficacy in treating PSD by inhibiting microglia activation (167), exerting anti-inflammatory effects (168), and reversing the decrease in neurogenesis induced by CMS (169). However, these findings await validation through rigorous randomized controlled trials in PSD patients.
- Neurotrophic factor and neurotransmitter modulators: Reduced levels of BDNF are a key link to impaired neurogenesis in PSD. Therefore, strategies to directly or indirectly enhance BDNF signaling are of considerable interest. Regarding interventions, basic research has explored various pathways: intravenous administration of BDNF after stroke enhances neuronal remodeling, resulting in improved functional outcomes including PSD (170). Several natural compounds have also been found to modulate BDNF. For instance, catapol, an antidiabetic, mediates adult hippocampal neurogenesis through the metabolic factor PI3K, thus acting as an effective agent for the treatment of PSD (171). Vitamin D could regulate 5-HT synthesis through tryptophan hydroxylase 2. Low serum vitamin D levels are associated with the development of PSD (172, 173). Additionally, stimulatory effects of vitamin D3 on the BDNF signaling pathway and neuroplasticity may play a role in improving PSD (174).
- Other neuroprotective agents and active herbal components: Several agents with multi-target neuroprotective effects show promise in PSD models. For example, Kim et al. treated PSD model mice with the phosphodiesterase-3 inhibitor Cilostazole, which reduced atrophy changes in the ipsilateral striatum and hippocampus by inhibiting neuron death and microglia activation, as well as enhancing neuronal differentiation (175). Furthermore, Kim et al. treated a mouse model of PSD with the antipsychotic aripiprazole, which induced the proliferation and differentiation of hippocampal NPCs, promoting neuroprotection and neurogenesis (176).

Traditional Chinese medicine (TCM) has accumulated substantial experience in treating PSD, and the mechanisms of its various active components have been elucidated by modern research. Gastrodin, the primary bioactive component of Gastrodia elata Blume, exhibits neuroprotective effects after ischemic injury by reducing microglial activity, modulating dopamine concentrations, increasing BDNF levels, and stimulating neurogenesis (177). Components such as morroniside and echinacoside can alleviate PSD symptoms by modulating miR-409-3p or Nrf2 acetylation to activate the BDNF/TrkB signaling pathway (178, 179). Research on herbal formulas also confirms that the Yinao Jieyu Formula can positively affect neurogenesis and improve depression-like behaviors in PSD mice by dynamically regulating the expression of genes in the Notch signaling pathway (180). In 2023, a meta-analysis showed that the combination of TCM (Shugan Jieyu capsule, Jie-Yu Pills, and Wuling capsule) with SSRI were among the most effective in terms of Hamilton depression scale score reduction response rates. The potential mechanisms may include promoting hippocampal neurorepair and neurogenesis, as well as improving synaptic plasticity (181).

### 4.2 Non-pharmacological and neuromodulatory interventions

#### 4.2.1 Repetitive transcranial magnetic stimulation

Neuroregulatory therapy has shown promise in treating PSD by employing electrical stimulation or pharmacological agents to activate specific targeted brain regions, thereby enhancing the function of the neurological system. In 2008, repetitive transcranial magnetic stimulation (rTMS), a novel non-invasive experimental technique, was approved by the FDA for the treatment of patients with MDD. A prospective randomized controlled trial suggested that high-frequency rTMS stimulation of the left dorsolateral prefrontal cortex could significantly improve depressive symptoms in the subacute phase of subcortical ischemic stroke (182). rTMS influences neuronal plasticity in the brain by generating long-term potentiation (LTP) and long-term depression (LTD), with the mechanisms of increasing BDNF levels, enhancing the synthesis and release of 5-HT, dopamine and norepinephrine, relieving inflammation response, regulating neurobiochemical processes, and promoting neuronal proliferation (183, 184). In 2020, Frey et al. demonstrated that rTMS is a safe and effective treatment for PSD (185). Low frequency TMS stimulate inhibitory neurons, while high frequency TMS stimulate excitatory projective neurons, simulating neuroplasticity through long-term enhancement (183).

#### 4.2.2 Other neuromodulation techniques

Electroconvulsive therapy (ECT) and transcranial direct current stimulation (tDCS), the other forms of neuroregulatory therapy, have limited data for the treatment of PSD. ECT, involved in regulating neurogenesis in the brain (186), has been regarded as the most powerful tool for the treatment of MDD (187). However, it is limited by related side effects such as memory loss (183). tDCS is a non-invasive method of stimulating the brain in which direct current is passed through areas that regulate mood and emotion, with aimed at regulating neural activity, thereby improving emotional balance. A trial involving 48 stroke patients evaluated the efficacy and safety of tDCS in the treatment of PSD. These patients receive tDCS treatments over a 6-week period, and the response and remission rates were significantly higher in the active group than in the sham group (188). Another randomized controlled trial with 50 acute stroke patients to determine the efficacy of tDCS on sensory and functional outcomes showed significant improvement in anxiety and depression symptoms within one year (189). Liu et al. used transcutaneous auricular vagus nerve stimulation(ta-VNS)combined with antidepressants to treat PSD, the results showed that ta-VNS may improve depressive symptoms by increasing serum 5-HT concentration and activating the BDNF-CREB signaling pathway (190).

#### 4.2.3 Exercise and integrative approaches

Aerobic exercise has well-established antidepressant and pro-neurogenic effects. In rodent models of PSD, exercise played an antidepressant role by regulating the relative levels of BDNF and proBDNF (191). A study demonstrated that Ninjin’yoeito, a traditional Japanese Kampo medicine, ameliorates serum corticosterone levels and hippocampal neuroinflammation in PSD rats. Furthermore, when combined with exercise therapy, Ninjin’yoeito exerts antidepressant effects by enhancing neurogenesis, the BDNF/proBDNF ratio, and mitigating neuroinflammation in the PSD hippocampus (192).

### 4.3 Future and experimental strategies: promises and translational challenges

This category encompasses promising but less-established interventions where clinical evidence is nascent and significant translational hurdles remain.

#### 4.3.1 Exogenous stem cell transplantation

NSCs/NPCs transplantation is an approach aimed at replacing lost neurons and triggering various mechanisms to alleviate depression symptoms. For instance, it induces the production of neurotrophic factors that promote cell survival or restore synaptic transmitter function through the integration of newborn neurons into existing neural network (193–195). The effect of intravenous NPCs on mice with PSD was assessed. The results showed that injection of NPCs improved depression-like behavior induced by ischemia, possibly by restoring BDNF-ERK-CREB signaling in the hippocampus. However, critical challenges for clinical application include ensuring targeted cell survival and functional integration in the hostile post-stroke environment, controlling potential immunogenic responses, and addressing ethical and practical sourcing of cells. The efficacy and safety profile in humans remain largely unknown, highlighting a significant gap between animal research and clinical therapy.

#### 4.3.2 Gut-brain axis modulation

The link between gut microbiota dysbiosis, inflammation, and depression has opened a novel therapeutic avenue. Preclinical studies demonstrate that probiotics or fecal microbiota transplantation can improve PSD rats’ neuronal function and depressive symptoms (196), possibly by modulating systemic inflammation and serotonin metabolism (197, 198). However, translating this to humans faces major challenges, including the high variability of human gut microbiota, the complexity of designing effective and standardized probiotic or transplant protocols, and a lack of large-scale RCTs confirming efficacy specifically for PSD.

#### 4.3.3 Epigenetic and miRNA-based therapies

Targeting epigenetic mechanisms (e.g., DNA methylation of the BDNF gene) or specific microRNAs that regulate neurogenic pathways represents a frontier in precision therapy (199). Feng et al. regulated the expression of vascular endothelial growth factor by knocking down the SET domain-containing 3, a histone H3 methyltransferase up-regulated in the PSD animal model (1). This regulation promoted the proliferation and differentiation of NSCs within the hippocampus, thereby improving depressive symptoms. Regulation of synaptic plasticity through related miRNA, which inhibit gene expression by binding to the 3UTR of mRNA, has received great attention in the treatment of MDD. Similarly, the direct targeting of these mRNAs, which regulates the gene expression of glucocorticoids, serotonin, and neurotrophins, show great potential in the management of PSD. However, the development of safe, brain-penetrant, and specific epigenetic drugs or miRNA delivery systems poses formidable technical and safety challenges, placing these strategies firmly in the experimental domain.

In summary, while pharmacological and established neuromodulatory treatments for PSD show clinical benefit with mechanisms linked to neurogenesis, their effects are indirect and multifaceted. The most innovative, direct pro-neurogenic strategies—such as cell therapy and precision microbiome/gene modulation—currently face substantial translational barriers. Future research must prioritize rigorous human trials for promising agents (e.g., specific neurotrophic modulators) and thoughtfully address the biological and technical challenges inherent in bringing advanced cellular and molecular therapies from bench to bedside for PSD patients.

## 5 Discussion and conclusions

PSD remains a significant and enduring challenge that adversely impacts the lives of stroke survivors. The pathophysiology of PSD is complex and multifactorial, arising from both neurobiological dysfunctions induced by ischemia and psychosocial distress. Among various neurobiological hypotheses, impaired hippocampal neurogenesis has emerged as a salient and central factor. This process involves the intricate integration of multiple systems, including hyperactivity of the HPA axis, neuroinflammation, reduced levels of neurotrophic factors (notably BDNF), dysregulation of monoaminergic and other neurotransmitters, and alterations in signaling pathways such as Notch.

While animal models, particularly those combining focal ischemia with CMS, have been indispensable in elucidating the link between hippocampal neurogenesis and PSD, critical limitations must be acknowledged. These models often simplify the profound psychosocial dimensions of human PSD, such as perceived loss, social role changes, and complex emotional coping. Furthermore, inherent differences in neuroanatomy, lifespan, and stress response systems between rodents and humans necessitate cautious extrapolation of findings. The specific timing, location, and severity of stroke, which vary greatly in patients, are also difficult to standardize in animal studies, potentially affecting the generalizability of neurogenic outcomes. Therefore, future research must prioritize bridging this translational gap.

To advance the field, several key lines of research should be prioritized. First, there is a pressing need for direct evidence in humans, utilizing advanced neuroimaging (e.g., PET ligands for neurogenesis markers) and post-mortem studies to confirm and quantify the role of hippocampal neurogenesis in PSD patients. Second, developing more sophisticated animal models that incorporate chronic psychosocial stressors more reflective of the human condition and that differentiate between stroke subtypes is crucial. Third, research should focus on elucidating the precise temporal sequence and causal interactions between the various mechanisms (HPA axis, inflammation, neurotrophic factors) in suppressing neurogenesis post-stroke. Finally, mechanism-driven therapeutic trials are needed, moving beyond broad neurogenesis promotion to target specific dysfunctional pathways (e.g., selective anti-inflammatory agents, HPA axis modulators, or BDNF enhancers) and rigorously evaluate their efficacy on both neurogenic and depressive outcomes in well-defined patient cohorts.

Recent studies have further elucidated the potential relationship between hippocampal neurogenesis and PSD while investigating the pharmacological mechanisms of antidepressant, many of which appear to exert their effects partly through promoting neurogenesis. Ongoing research into neural regulation and innovative pharmacotherapies aimed at promoting hippocampal neurogenesis continues to be essential. In conclusion, a strong interplay exists between PSD and hippocampal neurogenesis. Stroke-induced brain injury and the ensuing neurobiological alterations suppress hippocampal neurogenesis, which in turn contributes to and exacerbate depressive symptoms. Therefore, comprehensive interventions for PSD should encompass not only emotional management and social support but also proactive, mechanism-based strategies to promote neurogenesis and neuroplasticity, ultimately aiding patients in regaining functionality and enhancing their quality of life. In-depth, translationally focused research in this field will undoubtedly pave the way for novel preventative and therapeutic approaches to PSD.
